# *Paraphyton cookei* as a cause of dermatophytosis in calves

**DOI:** 10.1007/s11259-026-11619-0

**Published:** 2026-11-07

**Authors:** Matheus Mendes Pereira, Caroline Quintana Braga, Lara Baccarin Ianiski, Isabelle Carneiro Rizzatti, Henrique Zarnott Raatz, Yasmin Dummer Ruas, Luciéle Pereira Melo, Eliza Simone Viegas Sallis, Ana Lucia Pereira Schild, Sônia de Avila Botton, Daniela Isabel Brayer Pereira

**Affiliations:** 1 Self-employed professional, Santa Vitória do Palmar, RS Brazil; 2 Instituto de Biologia, Departamento de Microbiologia e Parasitologia, Laboratório de Micologia, Programa de Pós-Graduação em Microbiologia e Parasitologia, Universidade Federal de Pelotas (UFPel), Pelotas, RS Brazil; 3 Centro de Ciências da Saúde, Departamento de Microbiologia e Parasitologia, Laboratório de Pesquisas Micológicas, Programa de Pós-Graduação em Medicina Veterinária (PPGMV) e em Ciências Farmacêuticas (PPGCF), Universidade Federal de Santa Maria (UFSM), Santa Maria, RS Brazil; 4 Faculdade de Veterinária, Universidade Federal de Pelotas, Laboratório Regional de Diagnóstico, Pelotas, RS Brazil

**Keywords:** Bovine, Calves, Ringworm, Geophilic dermatophyte, Outbreak, Brazil

## Abstract

Dermatophytosis is a contagious and zoonotic superficial fungal infection affecting various animal species. In cattle production, ringworm has huge sanitary and economic relevance. Although *Trichophyton verrucosum* is the main etiological agent of bovine dermatophytosis, infections caused by other dermatophyte species may occasionally occur. This study reports an outbreak of dermatophytosis in calves caused by the geophilic dermatophyte *Paraphyton cookei* in southern Brazil. The outbreak occurred on a beef cattle farm in the state of Rio Grande do Sul during the spring. Of the 360 cattle in the herd, 120 were calves aged 60–90 days, of which 30 exhibited skin lesions characterized by irregular areas of alopecia of varying sizes, accompanied by small crusts and distributed across multiple body regions. Hair and crust samples were collected for mycological examination. Fungal culture produced colonies with macroscopic and microscopic characteristics consistent with *P. cookei*. The identification was further confirmed by sequencing of the internal transcribed spacer region of rDNA. To the best of our knowledge, this is the first report of dermatophytosis in calves caused by *P. cookei*. These findings highlight the importance of considering geophilic dermatophytes in the differential diagnosis of bovine dermatophytosis and reinforce the relevance of laboratory identification methods for accurate etiological diagnosis and epidemiological surveillance.

## Background

Dermatophytosis is a highly contagious cutaneous mycosis that affects keratinized tissues of humans and animals and represents an important sanitary and economic concern in livestock production, particularly in young cattle. The disease is characterized by infection of the skin and hair and is associated with economic losses due to hide damage, reduced productivity, treatment costs, and zoonotic risk (Chermette et al. 2008; Papini et al. 2009; Tartor et al. 2020; Pereira and Meireles 2023; Piorunek et al. 2024). In cattle herds, dermatophytosis usually occurs in an enzootic pattern or as outbreaks, especially under management conditions that favor transmission, such as animal crowding, intensive production systems, stress, poor hygiene, and the introduction of infected or carrier animals (Chermette et al. 2008; Tartor et al. 2020; Moskaluk and VandeWoude 2022; Pereira and Meireles 2023).

Dermatophytes belong to the family Arthrodermataceae and currently comprise nine genera, including *Trichophyton*, *Epidermophyton*, *Nannizzia*, *Paraphyton*, *Lophophyton*, *Microsporum*, *Arthroderma*, *Ctenomyces*, and *Guarromyces* (de Hoog et al. 2017).

Although *Trichophyton verrucosum* is widely recognized as the primary etiological agent of bovine dermatophytosis worldwide, other zoophilic and geophilic dermatophytes have occasionally been reported infecting food-producing animals (Papini et al. 2009; Łagowski et al. 2020; Pereira and Meireles 2023). This diversity highlights the importance of comprehensive etiological investigations, particularly during outbreaks, to ensure accurate diagnosis and a better understanding of the epidemiology of dermatophytosis in cattle.

*Paraphyton cookei*, previously classified as *Microsporum cookei* (de Hoog et al. 2017) is traditionally regarded as a geophilic dermatophyte widely distributed in the environment and frequently isolated from soil and from the hair of asymptomatic animals (Moraes and Almeida 1978; Ogórek et al. 2019; Gnat et al. 2022). This species has been considered to have low pathogenic potential; however, sporadic cases of infection in humans and animals have been described, indicating that it may behave as an opportunistic pathogen under specific conditions (Moraes and Almeida 1978; Caffara and Scagliarini 1999; Nardoni et al. 2016; Ogórek et al. 2019).

The objective of the present study was to report an outbreak of bovine dermatophytosis caused by *P. cookei* in southern Brazil.

## Case Presentation

**Fig. 1:**
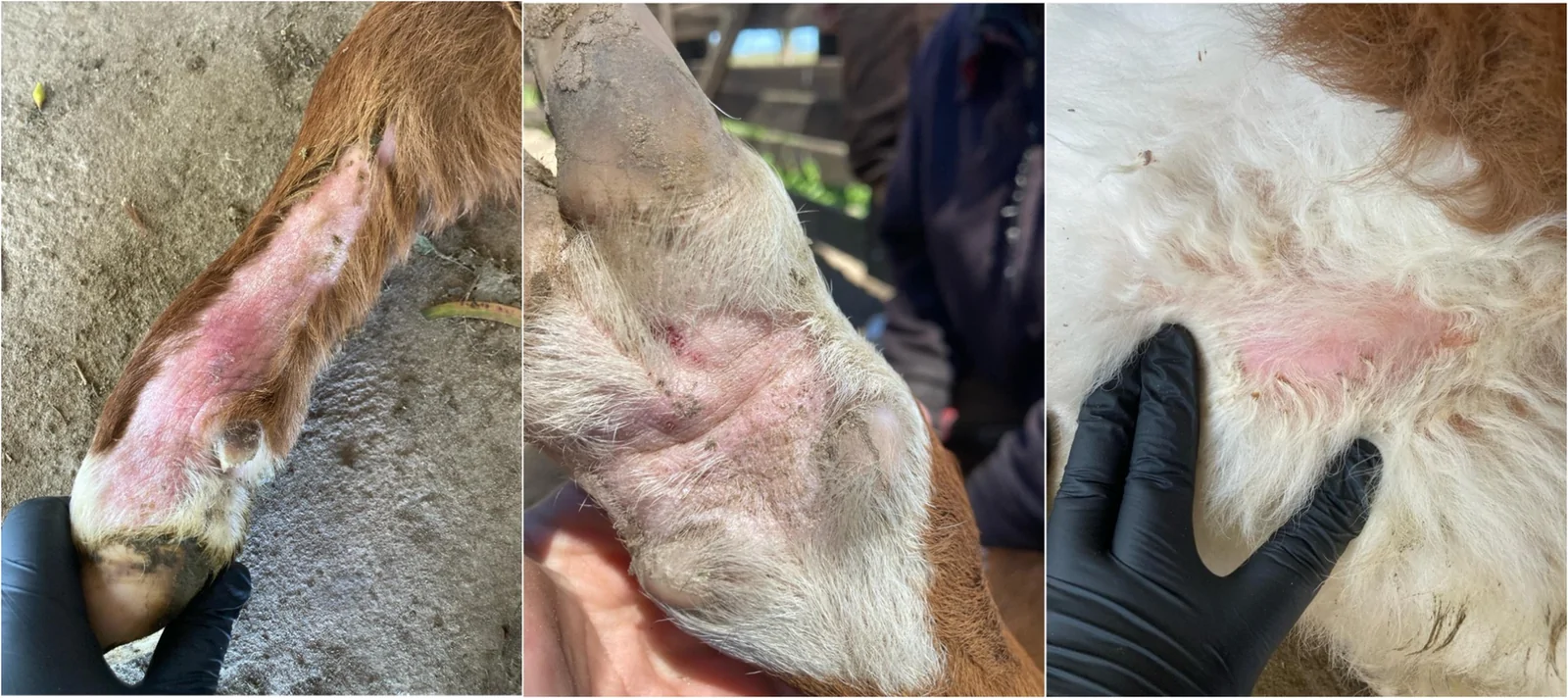
Bovine dermatophytosis caused by *Paraphyton cookei.* Areas of alopecia with marked dryness, accompanied by small crusts and mild superficial ulcerations, with erythema of the underlying skin

On a beef cattle farm located in the extreme south of Brazil (33° 31′ 08″ South latitude; 53° 22′ 04″ West longitude), in the state of Rio Grande do Sul, clinical signs consistent with dermatophytosis were observed in calves. The outbreak occurred during spring, in late September. The herd comprised 360 purebred Hereford and crossbred cattle, of which 120 were calves aged 60–90 days. Among these calves, which were suckling and raised extensively on ryegrass pasture, 30 developed irregular skin lesions of varying sizes. Clinical lesions were observed exclusively in this young age group, with no cases detected among adult cattle. The lesions were characterized by extensive areas of alopecia affecting multiple body sites, including the face, neck, limbs, and tail. The alopecic areas showed marked dryness, with the presence of small crusts and mild superficial ulcerations, leaving the underlying skin erythematous (Fig. 1). The affected calves received no antifungal treatment and showed clinical recovery within approximately 15 days, with no subsequent recurrence of lesions reported. In order to perform the laboratory diagnosis, hair and crust samples were collected from multiple skin lesions of a single affected animal and subjected to direct mycological examination using a 20% potassium hydroxide clarifying solution and cultured in Petri dishes containing Mycosel agar (Becton, Dickison and Company, USA), incubated at 25 °C for 21 days with daily observation. After the growth of colonies suspected to be dermatophytes, microculture and hair perforation tests were performed. The hair perforation test was carried out according to Ogórek et al. (2019) using *Trichophyton mentagrophytes* as the positive control. Species-level identification was performed through total DNA extraction according to Botton et al. (2011). Initially, the sample was cultured in an Erlenmeyer flask containing 100 mL of Sabouraud dextrose broth and incubated at 25 °C for seven days. After incubation, the mycelium was collected by filtration, placed in a microtube, and frozen at − 20 °C overnight. Subsequently, 600 µL of lysis solution [1.5% NaCl, 0.5% sodium dodecyl sulfate, 0.25 M ethylenediaminetetraacetic acid (EDTA) pH 8.0, 10 mM Tris-HCl pH 7.5, and 5 µL of 2-mercaptoethanol (Sigma-Aldrich, St. Louis, USA)] was added to the microtube, followed by 80 µL of 10% cetyltrimethylammonium bromide (CTAB) and 120 µL of 5 N sodium chloride (NaCl). The mixture was vigorously vortexed and incubated at 65 °C in a water bath for 45 min, followed by incubation at 37 °C for 15 min. Afterwards, 400 µL of ultrapure phenol (Invitrogen^®^) and 400 µL of chloroform: isoamyl alcohol (24:1, v/v) (Sigma-Aldrich, St. Louis, USA) were added, followed by further homogenization by vortexing until a homogeneous suspension was obtained. The sample was centrifuged at 13,500 rpm for 15 min, and 400 µL of the supernatant was transferred to a new microtube. An equivalent volume of isopropanol (Sigma-Aldrich, St. Louis, USA) was added, followed by gentle homogenization to precipitate the DNA. After centrifugation at 13,500 rpm for 15 min, the supernatant was discarded and the pellet (precipitate) was washed with 500 µL of 70% ethanol (Sigma-Aldrich, St. Louis, USA), followed by a new centrifugation at 13,500 rpm for 10 min. The ethanol was removed and the pellet was dried at room temperature. The DNA was resuspended in 100 µL of sterile TE buffer (10 mM Tris-HCl, pH 7.5; 1 mM EDTA, pH 8.0). Amplification of the ITS region was carried out by Polymerase Chain Reaction (PCR) using the primers ITS1 (5′-TCCGTAGGTGAACCTGCGG-3′) and ITS4 (5′-TCCTCCGCTTATTGATATGC-3′) (White et al. 1990). PCR reactions were performed in a PTC-100 thermocycler (Programmable Thermal Controller, MJ Research) under the following conditions: initial denaturation at 94 °C for 5 min; followed by 35 cycles consisting of denaturation at 94 °C for 30 s, annealing at 53 °C for 1 min, and extension at 72 °C for 2 min; with a final extension step at 72 °C for 5 min. The amplified products were verified by electrophoresis on 1% agarose gel stained with ethidium bromide and visualized under ultraviolet (UV) light. The PCR products were then purified using the PureLink PCR Purification Kit (Invitrogen^®^), and the purified sample was submitted for DNA sequencing. Sequencing of the PCR-amplified products was performed using an ABI Prism 3500 Genetic Analyzer automatic sequencer, employing the same primer pair used in the PCR reaction. The obtained sequences were analyzed using the BLAST tools (GenBank) to determine species identification based on the highest similarity index. Later, we performed a phylogenetic analysis based on the ITS region, including the bovine isolate (GenBank accession PQ124705.1), the type strain of *P. cookei* (CBS 228.58; NR_155665.1), and reference sequences of other *Paraphyton* species, including *P. mirabile* (IHEM 24409; HQ825145.1), *P. cookiellum* (CBS 101.83; MH861545.1), and *P. cutaneum* (UAMH 4027; MT192521.1), with *Trichophyton verrucosum* (CBS 365.53; NR_154982.1) as the outgroup. The phylogenetic tree was constructed using the Neighbor-Joining method based on uncorrected *p*-distances, with 500 bootstrap replicates.

### Microbiological findings

**Fig. 2:**
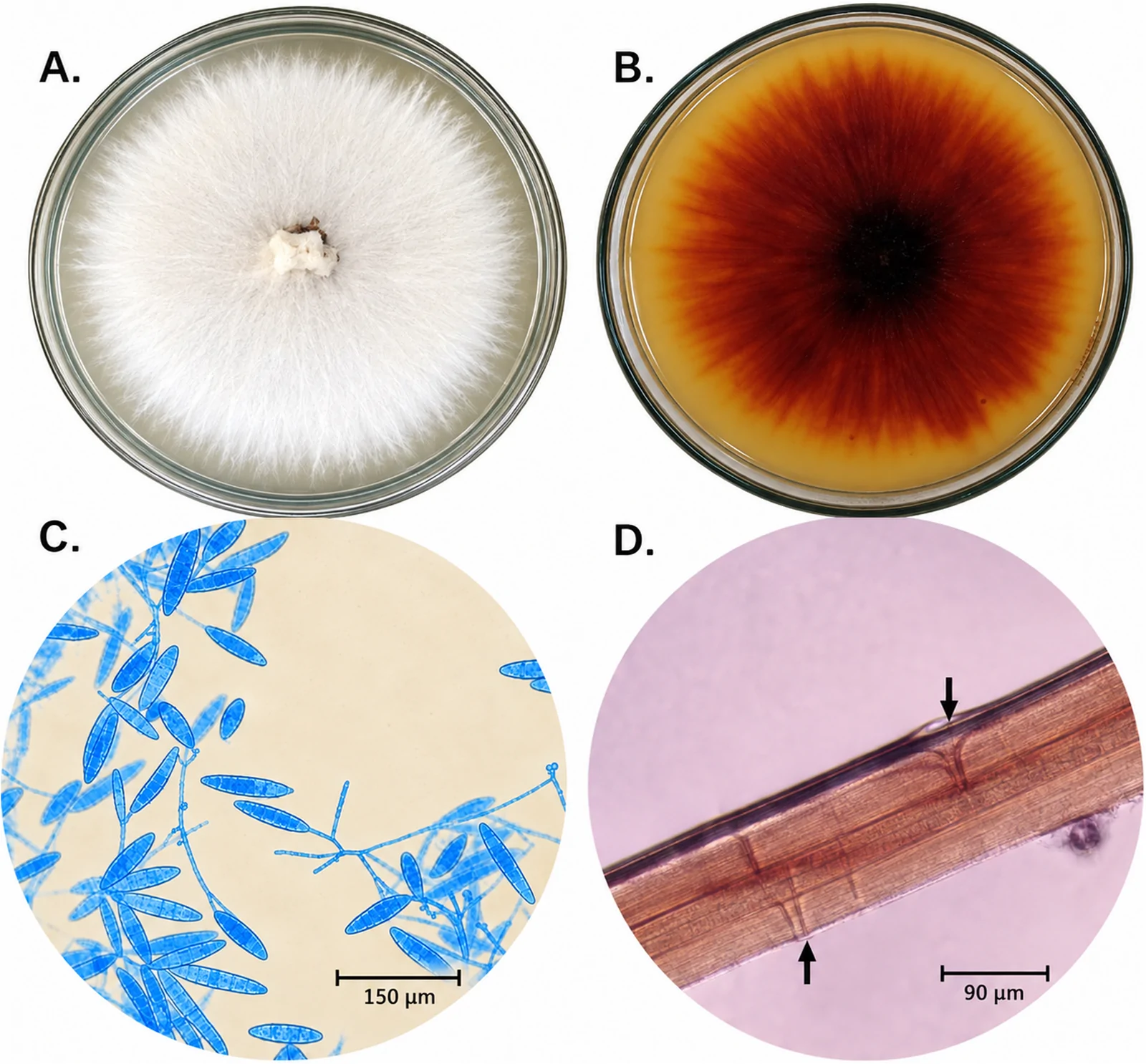
A and B. *Paraphyton cookei* culture on Mycosel agar, 21 days at 25 °C. (**A**) Observe appearance: colony with a suede-like color, powdery, granular appearance and radial grooves. (**B**) Reverse appearance: presence of reddish-purple to dark reddish-brown pigment. (**C**) Optical microscopy, with numerous large, elliptical macroconidia with very thick walls and echinulate (rough), with 5 to 6 septa (cotton blue, 40x objective). (**D**) Hair perforation test. Perpendicular wedge-shaped depressions are observed in the hair (arrow, 40x objective)

Direct examination of hair and crusts did not reveal parasitic fungal structures. However, after 21 days of incubation, granular and scattered colonies were observed, with coloration ranging from pale brown to suede-like, with a slightly raised and some radial grooves. The pigmentation on the reverse side of the culture medium varied from purplish-red to dark reddish-brown (Fig. 2A and B). Numerous large, very thick-walled, echinulate (rough) elliptical macroconidia with predominantly 5–6 septa were observed (Fig. 2C). The hair perforation test was positive and hair pitting appeared as perpendicular cotters (Fig. 2D).

**Fig. 3:**
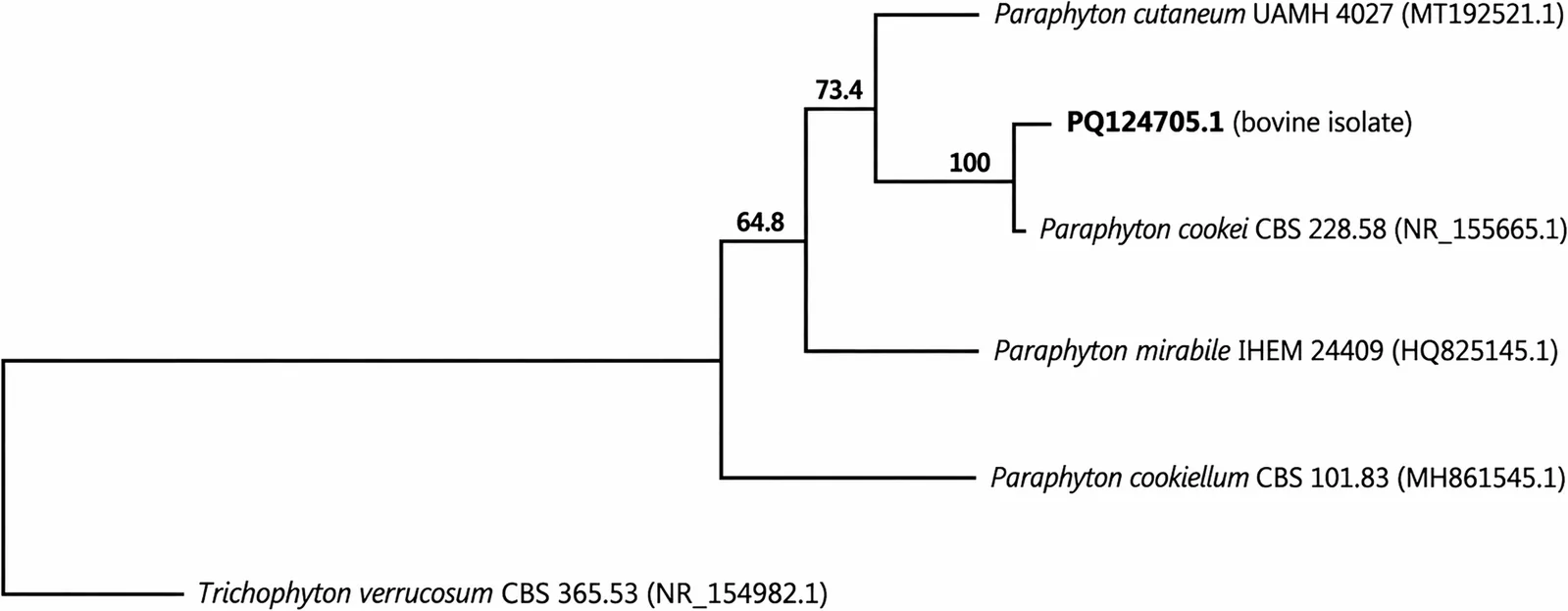
Phylogenetic analysis of the internal transcribed spacer (ITS) region showing the relationship between the bovine isolate (PQ124705.1) and reference strains of *Paraphyton* species. The tree was constructed using the Neighbor-Joining method based on uncorrected *p*-distances, with 500 bootstrap replicates. Bootstrap values (%) are indicated at the nodes. *Trichophyton verrucosum* (CBS 365.53) was used as the outgroup. The bovine isolate clustered with the type strain of *P. cookei* (CBS 228.58), with 100% bootstrap support

BLAST analysis of the ITS sequence obtained from the bovine isolate (GenBank accession PQ124705.1) revealed 100% nucleotide identity with previously deposited *P. cookei* sequences in GenBank. In addition, phylogenetic analysis based on the ITS region showed that the bovine isolate clustered with *P. cookei* type strain (CBS 228.58), with 100% bootstrap support. Pairwise sequence comparison between the bovine isolate and the type strain revealed 99.84% nucleotide identity, forming a distinct group separate from the other *Paraphyton* species examined (Fig. 3).

Microscopic characteristics, together with macroscopic observation of the culture and molecular identification, led to the identification of the dermatophyte as *P. cookei*. The sequence was deposited in GenBank under accession number PQ124705.

## Discussion and conclusions

Dermatophytosis in cattle is a disease of considerable veterinary and public health importance due to its zoonotic potential and the economic losses (Chermette et al. 2008; Papini et al. 2009; Tartor et al. 2020; Pereira and Meireles 2023; Piorunek et al. 2024). In addition, dermatophytosis is among the infectious skin diseases frequently diagnosed in cattle herds in southern Brazil, highlighting its relevance in regional livestock production systems (Silveira et al. 2003; Pereira and Meireles 2023; Venancio et al. 2024).

In the present report, an outbreak of dermatophytosis caused by the geophilic dermatophyte *P. cookei* occurred during spring, affecting 30 calves. Only young animals were affected, whereas adult cows maintained in the same environment remained clinically healthy. Dermatophytosis in cattle is typically enzootic and occurs more frequently in calves. The introduction of carrier animals, combined with stressful conditions such as rapid growth, weaning, parasitic burden, and nutritional deficiencies that compromise immunity and skin integrity, may favor the occurrence of the disease (Chermette et al. 2008; Papini et al. 2009; Tartor et al. 2020). In the present outbreak, age was likely the main predisposing factor. Calves aged 1–6 months, regardless of sex or breed, are more frequently affected than adult bovine because they have not yet developed immunity against dermatophytes. Additionally, young animals generally present a higher skin pH, which favors fungal growth and increases their susceptibility to infection (Moskaluk and VandeWoude 2022; Piorunek et al. 2024).

Bovine dermatophytosis is most commonly caused by *T. verrucosum* (Silveira et al. 2003; Chermette et al. 2008; Papini et al. 2009; Tartor et al. 2020; Pereira and Meireles 2023; Piorunek et al. 2024; Venancio et al. 2024). In Brazil, the disease has been reported in several regions, particularly in the state of Rio Grande do Sul, where outbreaks frequently occur during autumn and winter and may affect both young and adult animals (Silveira et al. 2003; Pereira and Meireles 2023; Venancio et al. 2024). Nevertheless, cases occurring in other seasons, including spring and summer, have also been described (Venancio et al. 2024), which is consistent with the seasonality observed in the present outbreak.

Other zoophilic and geophilic dermatophyte species, besides *T. verrucosum*, may occasionally infect cattle (Papini et al. 2009; Tartor et al. 2020; Łagowski et al. 2020; Piorunek et al. 2024). To the best of our knowledge, this is the first report of dermatophytosis in calves caused by *P. cookei*, a geophilic dermatophyte primarily associated with soil. The infections by this microorganism occur through environmental exposure and typically result in inflammatory but self-limiting lesions (Moraes and Almeida 1978; Ogórek et al. 2019; Gnat et al. 2022).

Here, the diagnosis of the disease was based on clinical findings, followed by direct microscopic examination of crusts and hair samples, as well as morphological and molecular identification of the fungal isolate obtained in culture. Clinically, the lesions observed in this outbreak consisted of extensive alopecic areas affecting multiple body sites, accompanied by small crusts, mild superficial ulcerations and underlying erythema. Dermatophytosis lesions arise from the invasion of keratinized tissues by dermatophytes and the subsequent release of proteolytic enzymes, which trigger inflammatory reactions leading to scaling, crust formation and hair loss (Moskaluk and VandeWoude 2022; Pereira and Meireles 2023).

Although the lesions observed in the present outbreak shared some similarities with those typically described in infections caused by *T. verrucosum*, subtle clinical differences were noted. In classical bovine ringworm caused by *T. verrucosum*, lesions usually appear as well-demarcated circular areas of alopecia with scaling and thick grayish crusts, predominantly affecting the head and neck and occasionally spreading to the trunk and limbs (Silveira et al. 2003; Chermette et al. 2008; Papini et al. 2009; Tartor et al. 2020; Pereira and Meireles 2023; Piorunek et al. 2024; Venancio et al. 2024). In contrast, such circumscribed grayish-white crusted lesions were not observed in this outbreak, in which lesions were mainly characterized by extensive alopecic areas. The clinical presentation of dermatophytosis caused by geophilic species may differ from that associated with zoophilic dermatophytes, typically inducing a more pronounced inflammatory response and a shorter course of infection (Moskaluk and VandeWoude 2022). These differences may explain the clinical pattern observed in the present outbreak and suggest a distinct presentation associated with infection by *P. cookei*, reinforcing the importance of laboratory identification for accurate etiological diagnosis in bovine dermatophytosis outbreaks.

Direct microscopic examination of crusts and hair failed to demonstrate fungal parasitism. Nevertheless, negative findings should be interpreted with caution, as inadequate sample collection may reduce the sensitivity of direct microscopy and hinder the detection of fungal elements in clinical specimens (Tartor et al. 2020). In contrast, the macroscopic and microscopic characteristics observed in culture were consistent with the phenotypic profile previously described for *P. cookei* (Nardoni et al. 2016; Ogórek et al. 2019; Piorunek et al. 2024), and species identification was subsequently confirmed by DNA sequencing.

*P. cookei* is widely distributed in nature and has been isolated from soil and from the hair of asymptomatic animals, indicating its ability to persist in the environment and participate in transmission cycles involving different hosts (Moraes and Almeida 1978; Ogórek et al. 2019; Gnat et al. 2022). This dermatophyte has low pathogenic potential, although sporadic infections in humans and animals have been reported, usually presenting mild clinical manifestations and spontaneous resolution within a few weeks (Moraes and Almeida 1978; Nardoni et al. 2016). Similarly, in the present outbreak, the affected calves showed clinical recovery within approximately 15 days without antifungal treatment.

Although antifungal susceptibility testing was not performed in our study, previously Gnat et al. (2022) evaluated four isolates recovered from asymptomatic European hedgehogs against 13 antifungal agents belonging to different classes, including allylamines, polyenes, imidazoles, triazoles, and other antifungal compounds. Terbinafine, luliconazole, amorolfine, and ciclopirox exhibited the lowest minimum inhibitory concentrations (MICs), indicating greater in vitro activity, whereas fluconazole and griseofulvin showed the highest MIC values. However, the limited number of isolates and the absence of established clinical breakpoints preclude definitive conclusions regarding antifungal susceptibility and therapeutic efficacy against *P. cookei* in naturally infected animals.

The limitations of this study are inherent to the investigation of a naturally occurring outbreak under field conditions. Although 30 calves exhibited clinical lesions consistent with dermatophytosis, mycological examination was performed on samples from only one affected animal, from which a single isolate of *P. cookei* was recovered and identified. Further sampling and epidemiological follow-up were not possible because the affected animals had already clinically recovered when additional investigation was requested. Furthermore, the recommended control measures were not implemented at the farm owner’s discretion, precluding an assessment of their potential impact on the epidemiological dynamics of the disease.

This study describes an outbreak of bovine dermatophytosis in southern Brazil in which *P. cookei* was identified in a single affected calf. Although the similar clinical manifestations and epidemiological characteristics observed among the affected animals suggest a possible association between this dermatophyte and the outbreak, its involvement in all cases could not be confirmed, as mycological investigations were limited to one animal. Nevertheless, this finding contributes to the limited knowledge of the potential involvement of *P. cookei* in bovine dermatophytosis and highlights the importance of considering uncommon dermatophytes in the differential diagnosis of bovine skin diseases. Furthermore, accurate laboratory identification of dermatophyte species is essential for improving epidemiological surveillance and for the effective prevention and control of dermatophytosis in livestock production systems.

## Data Availability

No datasets were generated or analysed during the current study.
